# O-arm–navigated open-door laminoplasty to reduce technical complications in multilevel cervical ossification of the posterior longitudinal ligament: operative video

**DOI:** 10.3171/2026.4.FOCVID2630

**Published:** 2026-07-01

**Authors:** Suchel Kim, Ryosuke Maruiwa, Keung Nyun Kim, Seong Yi, Dong-Ah Shin, Chang-Kyu Lee, Kota Watanabe, Yoon Ha

**Affiliations:** 1 Department of Neurosurgery, Severance Hospital, Yonsei University College of Medicine, Seoul, Republic of Korea; and; 2 Department of Orthopaedic Surgery, Keio University School of Medicine, Tokyo, Japan

**Keywords:** laminoplasty, O-arm navigation, ossification of the posterior longitudinal ligament, intraoperative navigation

## Abstract

Open-door laminoplasty is an established posterior decompression technique for multilevel cervical myelopathy. However, the procedure is technically demanding and may result in complications such as hinge fracture, facet joint violation, and improper gutter placement. The authors present an operative video demonstrating O-arm–based intraoperative navigation during open-door laminoplasty in a 75-year-old man with multilevel ossification of the posterior longitudinal ligament (OPLL) and severe cervical canal stenosis. Navigation allowed precise localization of the medial facet, controlled hinge preparation, and accurate screw placement. Postoperative CT confirmed adequate decompression without complications, suggesting that navigation may enhance the safety and precision of laminoplasty.

The video can be found here: https://stream.cadmore.media/r10.3171/2026.4.FOCVID2630

## Transcript

In this operative video, we demonstrate how O-arm–based intraoperative navigation can enhance surgical precision and reduce technical complications.

Open-door laminoplasty is a well-established posterior decompression technique for cervical myelopathy, particularly in patients with multilevel ossification of the posterior longitudinal ligament.

Despite its effectiveness, the procedure remains technically demanding. Inaccurate gutter placement,^1^ excessive hinge thinning, or improper screw trajectory may result in hinge fracture,^2^ facet joint violation,^3^ or insufficient decompression.^4^

### 1:04 Clinical Case.

A 75-year-old male presented with progressive lower extremity weakness and symptoms consistent with cervical myelopathy.

Preoperative MRI revealed severe spinal canal stenosis from C2 to C6 level due to multilevel OPLL.

At the C4–5 level, significant left-sided facet joint hypertrophy was noted, increasing the risk of improper gutter placement during conventional laminoplasty.^5^

An open-door laminoplasty was planned.

### 1:40 Preoperative Planning.

Preoperative CT imaging was reviewed to evaluate facet hypertrophy and laminar anatomy.

The average laminar thickness measured approximately 3.2 mm.

Based on these findings, the optimal gutter trajectory was carefully planned along the lamina–lateral mass junction while avoiding trajectory extension into the facet joint. The hinge side and open side was determined preoperatively to ensure safe preservation.

### 2:20 Patient Position and Setup.

The patient was positioned with the head secured in a three-pin Mayfield head holder. The navigation reference frame was attached to the Mayfield clamp. Navigation was established using intraoperative O-arm imaging.

To ensure the accuracy of cervical navigation, a screw was inserted at the tip of the spinous process and used intermittently as a reference marker to verify navigation accuracy.

### 2:57 Open-Side Gutter Creation.

Open-side gutter creation was performed under O-arm–based navigation.

Using real-time guidance, the medial border of the facet joint was clearly identified while gutter placement.

Navigation allowed precise localization of the lamina–lateral mass junction, which is the ideal site for gutter placement.

In cases of facet hypertrophy, conventional anatomical landmarks may be obscured or distorted.

Here, navigation provided anatomical confirmation, preventing inadvertent extension of the gutter into the facet joint.

The burr was advanced gradually and the depth of drilling was monitored continuously to ensure complete cortical penetration on the open side without overresection.

By confirming the exact orientation, we minimized the risk of improper gutter positioning.

### 4:07 Hinge Creation.

Hinge creation was performed on the contralateral side.

This step is critical, as excessive thinning may result in hinge fracture, whereas insufficient thinning may prevent adequate laminar opening.

Under O-arm navigation, drilling depth was assessed in real time.

The goal was to thin the dorsal cortex while preserving the ventral cortical layer to function as a greenstick hinge.

Navigation enabled visualization of the remaining cortical thickness, reducing the risk of overdrilling.

Particular care was taken to avoid creating a stress concentration point, which may result in intraoperative hinge fracture.

Symmetric thinning of the hinge zone facilitated predictable laminar opening.

### 5:17 Laminar Opening and Plate Fixation.

After bilateral gutters were prepared, gradual laminar opening was performed.

The lamina was elevated in gentle fashion to achieve adequate canal expansion.

Excessive force was avoided to prevent hinge disruption.

Following lamina opening, mini-plate fixation was applied to maintain the open-door configuration.

O-arm navigation was again utilized to determine optimal screw trajectory.

The dural edge on the open side was inspected to confirm the absence of injury.

And additional decompression was performed at C7 upper area.

### 6:20 Postoperative Imaging and Outcome.

Postoperative CT imaging confirmed adequate canal expansion from C2 to C6 level.

No hinge fracture was observed. There was no facet joint violation and improper gutter placement.

The patient experienced an uneventful postoperative course and was discharged without complications.

### 6:45 Discussion.

Although open-door laminoplasty is an effective decompression technique, it remains highly technique dependent.^6^

Complications such as hinge fracture, improper gutter positioning, and facet joint violation may occur when anatomical landmarks are distorted, particularly in multilevel OPLL with facet hypertrophy.

O-arm–based navigation provides real-time anatomical feedback, enabling precise gutter localization, controlled hinge preparation, and safe plate fixation.

### 7:26 Conclusion.

This technique is especially useful in cases with complex posterior element anatomy, where conventional visual landmarks may be unreliable.

By integrating intraoperative navigation, the safety and reproducibility of open-door laminoplasty may be enhanced.

And it can also reduce technical complications like hinge fracture, improper gutter position, and screw malposition.^7^

## Disclosures

The authors report no conflict of interest concerning the materials or methods used in this study or the findings specified in this publication.

## Author Contributions

Primary surgeon: Ha. Assistant surgeon: S Kim, Maruiwa. Editing and drafting the video and abstract: S Kim, Shin. Critically revising the work: S Kim, Lee. Reviewed submitted version of the work: Ha, KN Kim, Lee, Watanabe. Approved the final version of the work on behalf of all authors: Ha. Supervision: Ha, Yi, Shin.

## Supplemental Information

### Patient Informed Consent

The necessary patient informed consent was obtained in this study.
